# The Value of Nasal and Oral Clinical Examination in Febrile Neutropenic Patients for Initiating Antifungal Therapy as a Preemptive Method

**DOI:** 10.3389/fmed.2021.803600

**Published:** 2022-01-28

**Authors:** Mohammadreza Salehi, Sara Ghaderkhani, Ramezan Ali Sharifian, Seyed Ali Dehghan Manshadi, Elahe Samiee Fard, Sadegh Khodavaisy, Ramtin Pourahmad, Abbas Rahimi Foroushani, Kamran Rodini, Hasti Kamali Sarvestani

**Affiliations:** ^1^Department of Infectious Disease, Imam Khomeini Hospital Complex, Tehran University of Medical Sciences, Tehran, Iran; ^2^Department of Internal Medicine, Imam Khomeini Hospital Complex, Hematology and Oncology Ward, Tehran University of Medical Sciences, Tehran, Iran; ^3^Department of Medical Parasitology and Mycology, School of Public Health, Tehran University of Medical Sciences, Tehran, Iran; ^4^Faculty of Medicine, Tehran University of Medical Sciences, Tehran, Iran; ^5^Network of Immunity in Infection, Malignancy and Autoimmunity (NIIMA), Universal Scientific Education and Research Network (USERN), Tehran, Iran; ^6^Department of Epidemiology and Biostatistics, School of Public Health, Tehran University of Medical Sciences, Tehran, Iran

**Keywords:** invasive fungal infections, hematologic neoplasms, antifungal therapy, preemptive, empirical, neutropenia, nasal, oral

## Abstract

**Background:**

Invasive fungal infections (IFIs) are complications that lead to mortality and morbidity in hematologic malignancies. The time of starting antifungal therapy is vital. Preemptive antifungal therapy has appeared recently as a new policy for the management of IFIs based on noninvasive ways in neutropenic patients.

**Methods:**

We enrolled leukemia patients with neutropenia after chemotherapy in Imam Khomeini Hospital Complex, Tehran, Iran. Patients who entered the neutropenic phase were divided into two categories (empirical and preemptive) for receiving antifungal agents. The patients were clinically examined in the preemptive group every day to find IFIs. As soon as clinical evidence of IFIs was observed, antifungal was prescribed. The empirical group patients received antifungals based on the ward protocol. Based on the data in each group, the diagnostic and therapeutic results of cases are followed-up to 3 months. To compare percentages between the two groups, the chi-squared test was used. And to compare two means between the two groups, the independent *t*-test was used. All the statistical analyses were done in the Statistical Package for the Social Sciences (SPSS) version 24 software (IBM Corporation, Armonk, New York, USA).

**Results:**

We assessed 132 leukemic patients with inclusion and exclusion criteria. Eventually, 80 patients were enrolled. The mean age was 35.52 years. Demographics data and distribution of leukemia type show no significant differences between the two groups. Despite a higher percentage of IFIs discovered in the preemptive group than the empirical group (25 vs. 18.75%, respectively), but data show no significant differences. The average days of IFIs diagnosis since the beginning of neutropenia in the empirical group were 9.5 days while in the preemptive group, the average days were 5.4 days (*p* < 0.05). Totally, there were 15 patients with a proven IFI in each group (40% in the empirical group and 60% in the preemptive group). Results significantly show an increase in surgical sinus debridement in the empirical groups (83.3%) vs. the preemptive groups (55.5%), (*p* < 0.05). The mortality rate differed significantly among the two groups; it was 7.5% in the preemptive group and 25% in the empirical group (*p* < 0.05).

**Conclusion:**

Daily oral and nasal cavities examination to find the symptoms of IFIs and then start preemptive antifungal agents may be able to lead to accurate diagnosis, earlier treatment, and decreasing sinus surgery debridement in leukemia patients with neutropenia.

## Introduction

Invasive fungal infections (IFIs) are severe complications that lead to mortality and morbidity in patients with an impaired immune system, such as acute or chronic leukemia (1–3). Nowadays, we are facing a general increase in IFIs (4, 5). New chemotherapy medication causes longer neutropenia, which raises immunocompromised patients. The diagnosis of IFIs in the early stages is challenging and it is crucial for a better prognosis of antifungal treatments (6–8). According to a previous study, almost 33% of newly diagnosed acute myeloid leukemia (AML) under chemotherapy has developed IFIs. A 30-day mortality rate of IFIs among patients with AML in another study was about 22.1% (7, 9). The remarkable point is that cytotoxic chemotherapy as a treatment for leukemia causes critical neutropenia (10–12). Studies have shown that prolonged neutropenia is the most significant risk factor for getting IFIs (13–16). Clinical manifestations of IFIs in immunocompromised hosts can be atypical and silent; therefore, the diagnosis of fungal infections is troublous (15–17). Some symptoms, such as fever, facial pain, and headache are nonspecific (3, 18). IFIs in the nasal cavity represent pallor and discoloration, ischemia, necrosis, ulcer in the septum, or eschar in the early stages (18, 19). In contrast, the air-crescent sign in CT scan emerges lately with the existence of advanced IFI, which is not pleasing for early recognition (6). Recent studies proved that the incidence of IFIs is higher in patients with no empirical antifungal therapies (20–22).

*Candida* spp. are responsible for the most prevalent fungal nosocomial infections (23–25). IFIs can affect a single organ or spread across the body. Invasive candidiasis commonly affects the bloodstream. The lungs and sinuses are the typical sites for invasive aspergillosis (26–29).

There is an association between a long time and profound neutropenia with impaired prognosis in patients undergoing antifungal treatments (30, 31). The time of starting antifungal therapy is vital in high-risk patients including chemotherapy patients (32, 33). Based on previous studies, different centers perform antifungal therapies in various ways: empirical, preemptive, and targeted therapy (13, 34). Empirical antifungal therapy is one of the most accepted ones for patients with febrile neutropenia after 3–7 days of constant fever, despite broad-spectrum antibiotics (13, 35). Considering clinical manifestation and the risk of the patient for IFIs, it leads to initiating antifungal (empirical) therapy, regardless of microbiological therapy (36, 37).

Empirical antifungal therapy mostly causes overtreatment, drug resistance, higher medical costs, and is not dedicated (38, 39). Doubt about the advantages and disadvantages of empirical antifungal therapy created the necessity for making another strategy to help the patients (3, 40). Preemptive antifungal therapy has appeared recently as a new policy for the management of IFIs in neutropenic patients. This method uses noninvasive ways, such as imaging (CT scan) or serological tests (galactomannan testing), for starting antifungal agents in suspected neutropenic patients in IFIs. However, preemptive strategies may lead to the delayed beginning of antifungal therapies, have no effect on higher mortality in patients, and decrease the medication costs (41–43). A Chinese survey comparing empirical therapy to preemptive therapy demonstrated the same survival rate, but fewer changes for preemptive patients (43). Thus, we designed this study to evaluate the benefits of initiating preemptive antifungal therapy based on physical examination of oral and nasal cavities for finding the clinical features of IFIs as a complementary method in leukemic patients with neutropenia.

## Patients and Methods

### Study Population and Design

After evaluation of patients with acute leukemia, we enrolled hospitalized patients in Imam Khomeini Hospital Complex, Tehran, Iran from April 2018 to March 2020 who met the inclusion criteria in addition to none of the exclusion criteria and gave informed consent.

### Inclusion Criteria

Diagnosis of acute leukemia, receiving chemotherapy, and neutropenia defined as an absolute neutrophil count (ANC) of < 500 cells/mm^3^ were assumed as inclusion criteria. Peripheral blood smears of the leukemic patients were evaluated daily after chemotherapy for defining ANC.

### Exclusion Criteria

We considered exclusion criteria as follows: age < 14 years, septic shock, diagnosis of hematologic malignancy except for leukemia, history of prior invasive fungal infections, reluctance to participate in the project, and the impossibility of additional diagnostic tests for possible or definitive invasive fungal infection. Patients who entered the neutropenic phase were divided into two categories to have antifungal treatment approaches.

### Group 1

In the preemptive group, patients who entered the neutropenic phase (ANC < 500 cells/mm^3^) following chemotherapy without fever were daily examined to find a necrotic or ulcerative mucosal lesion, black scars, acute localized pain, purple discoloration in the nasal cavity, or similar lesions in the oral cavity, palate, and pharynx. We composed a datasheet that contains nasal and oral cavity examination features in the preemptive group. The daily examination was performed by a trained healthcare provider. The nasal speculum was used for nasal examination and evaluation of the mouth was performed by abeslang and a flashlight. As soon as discovering clinical evidence of invasive fungal infection (without starting fever), antifungal therapy with liposomal amphotericin B [3–5 mg/kg/daily/intravenous (IV)] was added to the medication regimen of the patient. Moreover, chest and paranasal CT scans, sinus endoscopy, and acquired samples were checked for pathology and culture and fungal smear. The serum galactomannan test was sent at the same time as seeing lesions in daily assessments. The endpoint for serial examination is recovery from neutropenia or the diagnosis of invasive fungal infection.

### Group 2

The empirical group includes patients who experienced fever in their neutropenic period subsequent chemotherapy. According to specified fever and neutropenia guidelines in the hematology department of Imam Khomeini Hospital Complex, cases were managed. Based on the mentioned protocol, after 3–5 days of fever and neutropenia and no response to broad-spectrum antibiotics—without severe sepsis or septic shock condition—liposomal amphotericin B (3–5 mg/kg/daily/IV) was started and diagnostic measurements, such as imaging and galactomannan level of the serum, were done.

All the patients with neutropenia in both groups received fluconazole 400 mg/PO/daily for antifungal prophylaxis. The clinical indicators assessed in clinical examinations were recorded in datasheets. The diagnosis of IFIs in this study was based on the European Organization for Research and Treatment of Cancer/Invasive Fungal Infections Cooperative Group (EORTC)/National Institute of Allergy and Infectious Diseases Mycoses Study Group (MSG) criteria (44). The definitions determined 3 levels of probability to the diagnosis of invasive fungal infection in immunocompromised patients including “proven,” “probable,” and “possible” invasive fungal infection.

“Proven” invasive fungal infection is diagnosed only when the presence of invasion by fungus can be identified by histological diagnosis or culture of a specimen taken from the site of infection. In contrast, “probable” and “possible” invasive fungal infections consist of 3 factors. Probable IFI requires a host factor, a clinical criterion, and mycological evidence. Patients with a host factor and a clinical criterion but without mycological evidence are considered as possible IFI (44, 45). The diagnosis of proven cases in this study was based on histopathological evidence or tissue fungal culture.

Finally, based on the data in each group, the diagnostic and therapeutic results of proven and probable IFI cases were followed and recorded until 3 months. To compare percentages between the two groups, the chi-squared test was used and for comparing two means between the two groups, the independent *t*-test was used. All the statistical analyses were done in the Statistical Package for the Social Sciences (SPSS) version 24 software (IBM Corporation, Armonk, New York, USA).

## Results

In this study, we assessed 132 leukemic patients referring to Imam Khomeini Hospital Complex in Tehran, Iran. They suffered from neutropenia after chemotherapy with inclusion and exclusion criteria. Eventually, 80 patients were enrolled. The empirical (control) group included 40 patients that developed a fever after chemotherapy. They were treated by antibiotic, if the treatment did not stop fever; patients were immediately given antifungal therapy by an empirical method. The preemptive (case) group consisted of 40 patients that underwent daily clinical nasal and oral examination.

Among 80 patients, the age range of cases was between 15 and 59 years and the average of all was 35.52 years, with 10.25 years SD. The type of leukemia is shown in Table 1. The chi-squared test showed that there is no significant association between type of malignancy and the treatment method (*p* > 0.05). Demographics data and leukemia type distribution show no significant differences between the two groups.

**Table 1:** Distribution of empirical and preemptive therapy in 80 leukemic patients.

**Type of malignancy, N (%)**	**Empirical**	**Preemptive**	**Total, N (%)**
AML^a^	29 (36.25%)	30 (37.50%)	59 (73.75%)
ALL^b^	11 (13.75%)	10 (12.50%)	21 (26.25%)
Total (%)	40 (50.00%)	40 (50.00%)	80 (100.00%)

a *AML, acute myeloid leukemia;*

b *ALL, acute lymphoblastic leukemia*.

### Incidence of Invasive Fungal Infections

According to the EORTC/MSG criteria for the classification of IFIs (44), results are shown in Table 2 divided by probable and proven cases in each group. The diagnosis of proven cases was based on histopathological evidence or tissue fungal culture. 12/15 (80%) cases were diagnosed based on histopathological evidence and 3/15 (20%) cases were diagnosed based on tissue fungal cultures as proven cases.

**Table 2:** Frequency of proven/probable fungal infections with regard to type of therapy.

		**Empirical**			**Preemptive**			**Total (%)**
Probable	IFIs positive, N (%)	9 (11.25%)			11 (13.75%)			
	IFIs negative, N (%)	31 (38.75%)			29 (36.25%)			
Proven	IFIs positive, N (%)	6 (7.50%)	Mucormycosis	3 (20.00%)	9 (11.25%)	Mucormycosis	5 (33.33%)	
			Aspergillosis	3 (20.00%)		Aspergillosis	4 (26.67%)	
	IFIs negative, N (%)	34 (42.50%)			31 (38.75%)			
Total (%)		40 (50.00%)			40 (50.00%)			80 (100%)

The average days of diagnosis of IFIs since the beginning of neutropenia in the empirical group were 9.5 days (with 2.01 days SD) while in preemptive patients, mean days of diagnosis were 5.4 days with 1.86 days SD and the difference in averages was statistically significant. The independent *t*-test showed a significant difference between the two means (*p* < 0.05).

Based on the results, nasal cavity symptoms were the dominant site (88.8%) of IFIs in this study, while 11.11% of patients had the symptoms of fungal infection in the oral cavity (change the color of the palate).

In total, out of 15 proven invasive fungal infections diagnosed in the two groups, 8 proven invasive fungal infections were mucormycosis and 7 proven invasive fungal infections were aspergillosis, while pathogen prevalence of IFIs is near the same in the two groups (Table 2). In each group, two patients had positive serum galactomannan levels (Table 3).

**Table 3:** Results of galactomannan test with regard to type of therapy.

**Galactomannan Test, N (%)**	**Empirical**		**N (%)**	**Preemptive**		**N (%)**	**Total (%)**
	Mucormycosis	Positive	0 (0.00%)	Mucormycosis	Positive	0 (0.00%)	0 (0.00%)
		Negative	3 (21.43%)		Negative	5 (35.71%)	8 (57.14%)
	Aspergillosis	Positive	2 (14.29%)	Aspergillosis	Positive	2 (14.29%)	4 (28.58%)
		Negative	1 (7.14%)		Negative	1 (7.14%)	2 (14.28%)
Total (%)			6 (42.86%)			8 (57.14%)	14 (100%)

There were 15 patients with a proven IFI and 6 (40%) patients related to the empirical group and 9 (60%) patients related to the preemptive group. In the course of the disease, 5 (83.33%) patients in the empirical group needed surgical sinus debridement. In contrast, among 9 preemptive patients, 5 (55.56%) patients needed debridement and 4 (44.44%) patients with IFIs did not need debridement.

In terms of the need for recurrent sinus surgery debridement, 83.3% of patients in the empirical group needed repetition, while this rate was 55.5% in the preemptive group. The chi-squared test significantly showed the increasing necessity of surgical sinus debridement of IFIs site in the empirical group vs. the preemptive group (*p* < 0.05).

The chi-squared test showed that all-cause mortality rate differed significantly among the two groups: lower mortality rate in the preemptive group against the empirical group. In the empirical group, 10 (25%) patients and in the preemptive group, 3 (7.5%) patients died in 3 months follow-up (*p* < 0.05).

## Discussion

Invasive fungal infection is one of the serious etiologies of chemotherapy-induced neutropenia (14, 45). As a common approach, empirical therapy has been used in treating IFI patients for years, but administering empirical antifungal agents may have certain problems, such as overtreatment or higher expenses and due to lack of trustworthy data, the efficiency of this method is still debatable (16, 38, 46).

With low specificity of clinical symptoms (e.g., fever, nodules, cough, hemoptysis, erythema, and maculopapular eruptions) and radiologic diagnostic tests, it has always been challenging to diagnose IFI in the early stages, but nowadays because of new diagnostic tools being employed for early identification of IFI, including the G-test, GM test, chest CT, and PCR, it is possible to define more exact starting points for antifungal treatment (4, 17, 18).

Studies comparing empirical vs. preemptive antifungal therapy in adult populations use different criteria including overall mortality, IFI-related mortality, percentage of patients with the final diagnosis of IFI, percentage of patients receiving antifungal therapy, and the number of days of antifungal treatment (19). To the best of our knowledge, this study is the first randomized controlled trial that recruits daily clinical nasal and oral examination before the manifestation of fever, as the inclusion criteria in the preemptive group and comparing with the empirical group. Patients with stem cell transplant and hematological disorders including neutropenic leukemia tend to have abrupt fatal courses of *Mucor spp*. or *Aspergillus spp*. sinusitis with a high mortality rate (47). Chen CY et al. reported 19 of 46 enrolled patients with invasive fungal sinusitis (IFS) died within 6 weeks indicating poor prognosis, especially in patients with prolonged neutropenia status in their study (ANC less than 500 cells/mm^3^ for more than 10 days) (48). During the daily nasal and oral examination, any symptom of nasal discharge, stuffiness, epistaxis, periorbital swelling, and maxillary tenderness as nonspecified and nose ulceration, eschar of the nasal mucosa, black necrotic lesions, and perforation of the hard palate as a more specific manifestation of IFS can lead to an early-stage diagnosis and antifungal therapy (47, 49, 50).

In this study, although numerically, the detection rate of fungal infections was higher in the preemptive group; this method could not significantly increase the diagnostic rate of invasive fungal infection in the preemptive group, which was 25% in the preemptive group vs. 18.75% in the empirical group (*p* > 0.05).

Lower rates of surgical sinus debridement in the preemptive group compared to the empirical group were needed. Surgical sinus debridement was done in 5/9 (55.56%) cases in the preemptive group and 5/6 (83.33%) cases in the empirical group (*p* < 0.05). On the other hand, the mortality rate differed significantly among the 2 groups with a noticeable reduction of mortality rate in the preemptive group (*p* < 0.05).

As mentioned in this study, the preemptive method that we devised was based on diagnosing IFI in neutropenic patients prior to the onset of fever, which differed from other studies that defined preemptive antifungal therapy as a strategy initiated after at least 4 days of refractory fever (38, 51, 52). This study shows a significant decrease in the average days of diagnosis of IFIs in the preemptive group (5.4 ± 1.86 days) vs. the empirical group (9.5 ± 2.01 days) that can indicate more efficiency of this study design to define preemptive therapy method in comparison with other studies (38, 56).

The mean age of patients was 35.52 years in our randomized controlled trial; As reported by Carol A Kauffman, these range of ages indulge more in social interactions and have more exposure to fungal and bacterial infections, so we can observe a higher incidence of histoplasmosis, aspergillosis, and cryptococcosis in these patients more than others (53). As reported in this study, the distribution of mucormycosis and aspergillosis in our population of interest with an age range of 35.52 years, with 10.25 years SD, was 8/15 (53.3%) and 7/15 (46.6%), respectively; that was in accordance to the prevalence of mentioned fungal infections reported by Njunda et al. (54) and Dhooria et al. (55) in their studied population.

Costs associated with antifungal therapy as an important reason for implementing preemptive antifungal therapy were indicated in a previous study by Cordonnier et al. (56) to lower the expenses of patients by 35% using the preemptive method. Although, they stated an incidence rate of IFI of 3/143 (9%) patients in the preemptive group vs. 4/150 (3%) patients in the empirical group that was in accordance with our IFI incidence rate. Ko et al. (35) also indicated the cost-effectiveness of a diagnostic-driven (preemptive) approach as a novel method to treat patients of hematological malignancies with neutropenia following chemotherapy and hematopoietic stem cell transplant (HCST) recipients with severe graft vs. host disease (GVHD). They also stated that choosing the preemptive or empirical therapy should be individualized according to the hospital and patient factors (e.g., feasibility, the turnaround time of diagnostics, local epidemiology, and risk of IFI). They suggested using preemptive strategy in the patients with low risk of IFI due to epidemiological factors or diagnostic tools and empirical strategy in high-risk patients or patients with severe illness.

According to the systematic review published in 2015, including 9 studies comparing preemptive vs. empirical antifungal therapy, it states that the medical expenses of leukemic neutropenia patients infected with IFI received preemptive antifungal strategy have shown significantly lower antifungal exposure and less clinical expenses than patients in the empirical group without increasing both the overall and IFI-related mortality rate (57). In another retrospective study, to evaluate preemptive antifungal strategy, 348 neutropenia episodes in 234 patients were reviewed. The main elements of preemptive therapy included a weekly chest CT scan and GM test twice a week. Patients were under prophylactic therapy with fluconazole 400 mg and antifungal therapy started at the stage of prognosis of IFI. Chest CT scans in a 10-day interval in 81% of cases diagnosed 109 patients with IFI. Forty-nine patients passed away before day 100 and 2 IFI cases were found after their death that was not diagnosed in preemptive antifungal strategy.

As reported in studies and protocols, in the majority of cases, it takes 3–7 days of persistent fever to start antifungal therapy in empirical method and obviously, there is a high risk of disseminated infection, but in the preemptive approach with our novel study design, we can observe a significant decrease in IFI-related and overall mortality (13, 35, 57). This study shares the same results on the early-stage diagnosis of IFI but additionally, despite many previous studies, we can observe a decrease in mortality rate in the preemptive group compared to the empirical group.

This randomized study revealed that preemptive antifungal treatment guided by daily nasal and oral examination before the manifestation of fever in addition to imaging findings and the GM test was able to significantly decrease the mortality rate in the preemptive group with decreasing the need for surgical debridement in leukemia patients with neutropenia.

## Conclusion

Daily oral and nasal cavities examination to find the features of IFIs and then start preemptive antifungal agents may be able to lead to accurate diagnosis, earlier treatment, decreasing sinus surgery debridement, and even mortality in leukemia patients with neutropenia.

## Data Availability Statement

The original contributions presented in the study are included in the article/supplementary material, further inquiries can be directed to the corresponding author.

## Ethics Statement

The studies involving human participants were reviewed and approved by Tehran University of Medical Sciences, Tehran, Iran. The patients/participants provided their written informed consent to participate in this study.

## Author Contributions

All authors listed have made a substantial, direct, and intellectual contribution to the work and approved it for publication.

## Funding

This study was supported by funding from the Tehran University of Medical Sciences, Tehran, Iran.

## Conflict of Interest

The authors declare that the research was conducted in the absence of any commercial or financial relationships that could be construed as a potential conflict of interest.

## Publisher's Note

All claims expressed in this article are solely those of the authors and do not necessarily represent those of their affiliated organizations, or those of the publisher, the editors and the reviewers. Any product that may be evaluated in this article, or claim that may be made by its manufacturer, is not guaranteed or endorsed by the publisher.

## References

[1] Torres-Flores J Espinoza-Zamora R Garcia-Mendez J Cervera-Ceballos E Sosa-Espinoza A Zapata-Canto N. Treatment-Related mortality from infectious complications in an acute leukemia clinic. J Hematol. (2020) 9:123–31. 10.14740/jh751 33224392 PMC7665858

[2] Ascioglu S Rex JH de Pauw B Bennett JE Bille J Crokaert F. Defining opportunistic invasive fungal infections in immunocompromised patients with cancer and hematopoietic stem cell transplants: an international consensus. Clin Infect Dis. (2002) 34:7–14. 10.1086/323335 11731939

[3] Mikolajewska A Schwartz S Ruhnke M. Antifungal treatment strategies in patients with haematological diseases or cancer: from prophylaxis to empirical, pre-emptive and targeted therapy. Mycoses. (2012) 55:2–16. 10.1111/j.1439-0507.2010.01961.x 21554421

[4] Schmiedel Y Zimmerli S. Common invasive fungal diseases: an overview of invasive candidiasis, aspergillosis, cryptococcosis, and Pneumocystis pneumonia. Swiss Med Wkly. (2016) 146:w14281. 10.4414/smw.2016.14281 26901377

[5] Bitar D Lortholary O Strat Y Nicolau J Coignard B Tattevin P. Population-based analysis of invasive fungal infections, France, 2001–2010. Emerg Infect Dis. (2014) 20:1149. 10.3201/eid2007.140087 24960557 PMC4073874

[6] Caillot D Couaillier JF Bernard A Casasnovas O Denning DW Mannone L. Increasing volume and changing characteristics of invasive pulmonary aspergillosis on sequential thoracic computed tomography scans in patients with neutropenia. J Clin Oncol. (2001) 19:253–9. 10.1200/JCO.2001.19.1.253 11134220

[7] Bassetti M Trecarichi EM Righi E Sanguinetti M Bisio F Posteraro B. Incidence, risk factors, and predictors of outcome of candidemia. Survey in 2 Italian university hospitals. Diagn Microbiol Infect Dis. (2007) 58:325–31. 10.1016/j.diagmicrobio.2007.01.005 17350205

[8] Garey KW Rege M Pai MP Mingo DE Suda KJ Turpin RS. Time to initiation of fluconazole therapy impacts mortality in patients with candidemia: a multi-institutional study. Clin Infect Dis. (2006) 43:25–31. 10.1086/504810 16758414

[9] Lien MY Chou CH Lin CC Bai LY Chiu CF Yeh SP. Epidemiology and risk factors for invasive fungal infections during induction chemotherapy for newly diagnosed acute myeloid leukemia: a retrospective cohort study. PLoS ONE. (2018) 13:e0197851. 10.1371/journal.pone.0197851 29883443 PMC5993235

[10] Netelenbos T Massey E de Wreede LC Harding K Hamblin A Sekhar M. The burden of invasive infections in neutropenic patients: incidence, outcomes, and use of granulocyte transfusions. Transfusion. (2019) 59:160–8. 10.1111/trf.14994 30383912 PMC7379528

[11] Schwartzberg LS. Neutropenia: etiology and pathogenesis. Clin Cornerstone. (2006) 8:S5–S11. 10.1016/S1098-3597(06)80053-0 17379162

[12] Crawford J Dale DC Lyman GH. Chemotherapy-induced neutropenia: risks, consequences, and new directions for its management. Cancer. (2004) 100:228–37. 10.1002/cncr.11882 14716755

[13] Leventakos K Lewis RE Kontoyiannis DP. Fungal infections in leukemia patients: how do we prevent and treat them? Clin Infect Dis. (2010) 50:405–15. 10.1086/649879 20047485

[14] Ramón O Bueno J. Fungal infections in neutropenic patients. Sangre. (1995) 40:17–23. 7716666

[15] Lamoth F Calandra T. Early diagnosis of invasive mould infections and disease. J Antimicrob Chemother. (2017) 72:i19–i28. 10.1093/jac/dkx030 28355464

[16] Lackner M Lass-Flörl C. Up-date on diagnostic strategies of invasive aspergillosis. Curr Pharm Des. (2013) 19:3595–614. 10.2174/13816128113199990323 23278540

[17] Latgé JP Chamilos G. Aspergillus fumigatus and Aspergillosis in 2019. Clin Microbiol Rev. (2019) 33:e00140-18. 10.1128/CMR.00140-18 31722890 PMC6860006

[18] Singh V. Fungal rhinosinusitis: unravelling the disease spectrum. J Maxillofac Oral Surg. (2019) 18:164–79. 10.1007/s12663-018-01182-w 30996535 PMC6441414

[19] Barrs VR Beatty JA Dhand NK Talbot JJ Bell E Abraham LA. Computed tomographic features of feline sino-nasal and sino-orbital aspergillosis. Veterinary J. (2014) 201:215–22. 10.1016/j.tvjl.2014.02.020 24685469

[20] Ruhnke M. Epidemiology of Candida albicans infections and role of non-Candida-albicans yeasts. Curr Drug Targets. (2006) 7:495–504. 10.2174/138945006776359421 16611037

[21] Hoenigl M Zollner-Schwetz I Sill H Linkesch W Lass-Flörl C Schnedl W. Epidemiology of invasive fungal infections and rationale for antifungal therapy in patients with haematological malignancies. Mycoses. (2011) 54:454–9.? 10.1111/j.1439-0507.2010.01881.x 20406398

[22] Kamali Sarvestani H Daie Ghazvini R Hashemi SJ Gerami Shoar M Ansari S Rafat Z. Molecular characterization of fungal colonization on the Provax in post laryngectomy patients. J Public Health. (2022) 51:151–9. 10.18502/ijph.v51i1.8306 PMC8837888 35223636

[23] Pagano L Caira M Candoni A Offidani M Fianchi L Martino B. The epidemiology of fungal infections in patients with hematologic malignancies: the SEIFEM-2004 study. Haematologica. (2006) 91:1068–75. 16885047

[24] Snydman David R. Shifting patterns in the epidemiology of nosocomial Candida infections. Chest. (2003) 123:500S−503S. 10.1378/chest.123.5_suppl.500S 12740235

[25] Bhatt VR Viola GM Ferrajoli A. Invasive fungal infections in acute leukemia. Ther Adv Hematol. (2011) 2:231–47. 10.1177/2040620711410098 23556092 PMC3573411

[26] Perusquía-Ortiz AM Vázquez-González D Bonifaz A. Opportunistic filamentous mycoses: aspergillosis, mucormycosis, phaeohyphomycosis and hyalohyphomycosis. J Dtsch Dermatol Ges. (2012) 10:611–21; quiz 21–2. 10.1111/j.1610-0387.2012.07994.x 22925358

[27] Patterson TF Thompson GR Denning DW Fishman JA Hadley S Herbrecht R. Practice guidelines for the diagnosis and management of aspergillosis: 2016 update by the infectious diseases Society of America. Clin Infect Dis. (2016) 63:e1–e60. 10.1093/cid/ciw326 27365388 PMC4967602

[28] Legouge C Caillot D Chrétien ML Lafon I Ferrant E Audia S. The reversed halo sign: pathognomonic pattern of pulmonary mucormycosis in leukemic patients with neutropenia? Clin Infect Dis. (2014) 58:672–8. 10.1093/cid/cit929 24352351

[29] Rausch CR DiPippo AJ Bose P Kontoyiannis DP. Breakthrough fungal infections in patients with leukemia receiving isavuconazole. Clin Infect Dis. (2018) 67:1610–3. 10.1093/cid/ciy406 29771293 PMC6454429

[30] Telles DR Karki N Marshall MW. Oral fungal infections: diagnosis and management. Dent Clin North Am. (2017) 61:319–49. 10.1016/j.cden.2016.12.004 28317569

[31] Ruhnke M Cornely OA Schmidt-Hieber M Alakel N Boell B Buchheidt D. Treatment of invasive fungal diseases in cancer patients-Revised 2019 Recommendations of the Infectious Diseases Working Party (AGIHO) of the German Society of Hematology and Oncology (DGHO). Mycoses. (2020) 63:653–82. 10.1111/myc.13082 32236989

[32] Posteraro B Tumbarello M De Pascale G Liberto E Vallecoccia MS De Carolis E. (1,3)-β-d-Glucan-based antifungal treatment in critically ill adults at high risk of candidaemia: an observational study. J Antimicrob Chemother. (2016) 71:2262–9. 10.1093/jac/dkw112 27125554

[33] Heinz WJ Buchheidt D Christopeit M von Lilienfeld-Toal M Cornely OA Einsele H. Diagnosis and empirical treatment of fever of unknown origin (FUO) in adult neutropenic patients: guidelines of the Infectious Diseases Working Party (AGIHO) of the German Society of Hematology and Medical Oncology (DGHO). Ann Hematol. (2017) 96:1775–92. 10.1007/s00277-017-3098-3 28856437 PMC5645428

[34] Tan BH Low JG Chlebicka NL Kurup A Cheah FK Lin RT. Galactomannan-guided preemptive vs. empirical antifungals in the persistently febrile neutropenic patient: a prospective randomized study. Int J Infect Dis. (2011) 15:e350–6. 10.1016/j.ijid.2011.01.011 21397541

[35] Ko BS Chen WT Kung HC Wu UI Tang JL Yao M. 2016 guideline strategies for the use of antifungal agents in patients with hematological malignancies or hematopoietic stem cell transplantation recipients in Taiwan. J Microbiol Immunol Infect. (2018) 51:287–301. 10.1016/j.jmii.2017.07.005 28781151

[36] Schneider T Halter J Heim D Passweg J Stern M Tichelli A. Pre-emptive diagnosis and treatment of fungal infections–evaluation of a single-centre policy. Clin Microbiol Infect. (2012) 18:189–94. 10.1111/j.1469-0691.2011.03589.x 21729194

[37] Rüping Maria JGT Jörg J Vehreschild Oliver A. Cornely. Patients at high risk of invasive fungal infections: when and how to treat. Drugs. (2008) : 68:1941–62. 10.2165/00003495-200868140-00002 18778118

[38] Yuan W Ren J Guo X Guo X Cai S. Preemptive antifungal therapy for febrile neutropenic hematological malignancy patients in China. Med Sci Monit. (2016) 22:4226–32. 10.12659/MSM.897596 27819257 PMC5110226

[39] de Pauw BE. Between over- and undertreatment of invasive fungal disease. Clin Infect Dis. (2005) 41:1251–3. 10.1086/496933 16206098

[40] Segal B Almyroudis N Battiwalla M Herbrecht R Perfect J Walsh T. Prevention and early treatment of invasive fungal infection in patients with cancer and neutropenia and in stem cell transplant recipients in the era of newer broad-spectrum antifungal agents and diagnostic adjuncts. Clin Infect Dis. (2007) 44:402–9. 10.1086/510677 17205448

[41] Ardi P Daie-Ghazvini R Hashemi SJ Salehi MR Bakhshi H Rafat Z. Study on invasive aspergillosis using galactomannan enzyme immunoassay and determining antifungal drug susceptibility among hospitalized patients with hematologic malignancies or candidates for organ transplantation. Microb Pathogenesis. (2020) 147:104382. 10.1016/j.micpath.2020.104382 32663605

[42] Blot SI Taccone FS Van den Abeele AM Bulpa P Meersseman W Brusselaers N. A clinical algorithm to diagnose invasive pulmonary aspergillosis in critically ill patients. Am J Respir Crit Care Med. (2012) 186:56–64. 10.1164/rccm.201111-1978OC 22517788

[43] Chen K Wang Q Pleasants RA Ge L Liu W Peng K. Empiric treatment against invasive fungal diseases in febrile neutropenic patients: a systematic review and network meta-analysis. BMC Infectious Diseases. (2017) 17:159. 10.1186/s12879-017-2263-6 28219330 PMC5319086

[44] De Pauw B Walsh Th Donnelly J Stevens D Edwards J Calandra T. Revised definitions of invasive fungal disease from the European organization for research and treatment of cancer/invasive fungal infections cooperative group and the national institute of allergy and infectious diseases mycoses study group (EORTC/MSG) consensus group. Clin Infect Dis. (2008) 46:1813–21. 10.1086/588660 18462102 PMC2671227

[45] Monsereenusorn Ch Sricharoen Th Rujkijyanont P Suwanpakdee D Photia A Lertvivatpong N. Clinical characteristics and predictive factors of invasive fungal disease in pediatric oncology patients with febrile neutropenia in a country with limited resources. Pediatr Health Med Therapeutics. (2021) 12:335. 10.2147/PHMT.S299965 34285630 PMC8285294

[46] Deng Q Lv H Lin X Zhao M Geng L Li Y. Empirical antifungal treatment for diagnosed and undiagnosed invasive fungal disease in patients with hematologic malignancies. Curr Med Res Opin. (2018) 34:1209–16. 10.1080/03007995.2017.1386167 28956459

[47] Bethge WA Schmalzing M Stuhler G Schumacher U Kröber SM Horger M. Mucormycoses in patients with hematologic malignancies: an emerging fungal infection. Haematologica. (2005) 90 Suppl:Ecr22. 16266913

[48] Chen CY Sheng WH Cheng A Chen YC Tsay W Tang JL. Invasive fungal sinusitis in patients with hematological malignancy: 15 years experience in a single university hospital in Taiwan. BMC Infect Dis. (2011) 11:250. 10.1186/1471-2334-11-250 21939544 PMC3196720

[49] Howells RC Ramadan HH. Usefulness of computed tomography and magnetic resonance in fulminant invasive fungal rhinosinusitis. Am J Rhinol. (2001) 15:255–61. 10.1177/194589240101500407 11554658

[50] Nosari A Oreste P Montillo M Carrafiello G Draisci M Muti G. Mucormycosis in hematologic malignancies: an emerging fungal infection. Haematologica. (2000) 85:1068–71. 11025599

[51] Maertens J Theunissen K Verhoef G Verschakelen J Lagrou K Verbeken E. Galactomannan and computed tomography-based preemptive antifungal therapy in neutropenic patients at high risk for invasive fungal infection: a prospective feasibility study. Clin Infect Dis. (2005) 41:1242–50. 10.1086/496927 16206097

[52] Morrissey CO Slavin MA. Antifungal strategies for managing invasive aspergillosis: the prospects for a pre-emptive treatment strategy. Med Mycol. (2006) 44:S333–S48. 10.1080/13693780600826699 30408927

[53] Kauffman CA. Fungal infections in older adults. Clin Infect Dis. (2001) 33:550–5. 10.1086/322685 11462194

[54] Njunda A Nsagha D Assob J Abange B Tamoh A Kwenti T. Pulmonary paragonimiasis and aspergillosis in patients suspected of tuberculosis in yaounde, cameroon. Microbiol Res J Int. (2015) 10:1–8. 10.9734/BMRJ/2015/20138

[55] Dhooria S Kumar P Saikia B Aggarwal AN Gupta D Behera D. Prevalence of Aspergillus sensitisation in pulmonary tuberculosis-related fibrocavitary disease. Int J Tuberc Lung Dis. (2014) 18:850–5. 10.5588/ijtld.13.0838 24902565

[56] Cordonnier C Pautas C Maury S Vekhoff A Farhat H Suarez F. Empirical versus preemptive antifungal therapy for high-risk, febrile, neutropenic patients: a randomized, controlled trial. Clin Infect Dis. (2009) 15;48:1042–51. 10.1086/597395 19281327

[57] Goldberg E Gafter-Gvili A Paul M Robenshtok E Vidal L Leibovici L. Empirical antifungal therapy for patients with neutropenia and persitent fever - systematic review and meta-analysis. Blood. (2007) 110:4961. 10.1182/blood.V110.11.4961.4961 18706808

